# Prevalence and factors associated with early postnatal care utilization among women of reproductive age in Tanzania: analysis of Tanzania demographic health survey 2015/16

**DOI:** 10.11604/pamj.2024.47.163.34368

**Published:** 2024-04-04

**Authors:** Hamidu Adinani, Caroline Amour, Sia Msuya, Cecilia S Anthony, Modesta Mitao, Winfrida Mwita, Jenny Renju

**Affiliations:** 1 Institute of Public Health, Department of Epidemiology and Biostatistics, Kilimanjaro Christian Medical University College (KCMUCo), PO. Box 2240, Moshi, Tanzania,; 2 Department of Population Health London School of Hygiene and Tropical Medicine Keppel St, London, England

**Keywords:** Postnatal care, early postnatal checkup, women of reproductive age

## Abstract

**Introduction:**

postnatal care (PNC) is critical for the health and survival of the mother and the newborn. The timing of the first postnatal checkup is crucial for the early identification and treatment of complications. Late or zero attendance of postnatal checkups negatively influences the health of the mother and the newborn. The study’s purpose is to determine the prevalence and factors associated with early postnatal care utilization among women of reproductive age (WRA) in Tanzania.

**Methods:**

this is an analytical cross-sectional study, using Tanzania demographic health survey data for 2015/16. Women of reproductive age (15-49 years) who gave birth 5 years prior the survey were analyzed. Data analysis was performed using Stata software Version 15. The Poisson regression analysis was used to assess factors associated with early PNC.

**Results:**

the prevalence of early PNC utilization in Tanzania was 36%. The identified determinants for early PNC were geographical zone, place of residence, access to media, place of delivery and mode of delivery. The prevalence of early PNC was higher among mothers with access to media, with caesarian delivery and to those with facility delivery. The prevalence was low among mothers who lived in rural areas, from southwest and lake zones.

**Conclusion:**

the coverage of early PNC was found to be low in Tanzania. Interventions informed by the identified factors need to be designed and implemented to improve the coverage of early PNC.

## Introduction

In 2017, World Health Organization (WHO) estimated that more than five million children worldwide died before their fifth birthday, of which 2.5 million deaths were neonatal deaths [1]. The majority (79%) of the neonatal deaths occurred in sub-Saharan Africa and Southern Asia, West and Central Africa accounted 23% of neonatal deaths, east and South Africa accounts 18% of neonatal deaths [2]. In the same year (2017) an estimated 295,000 women died worldwide due to maternal related causes, of which 86% (254,000) of the deaths occurred in developing countries, and 66% (196,000) were in sub-Saharan Africa [1]. In Africa, every year at least 125,000 women and 870,000 newborns die in the first week after birth while 50% of maternal deaths and 40% of neonatal deaths occur in the first 24 hours of life [3,4]. The leading cause of maternal mortality in developing countries include postpartum hemorrhage, hypertensive disorders and puerperal sepsis, while perinatal asphyxia and neonatal infections are the common causes of neonatal deaths [5]. In Tanzania, maternal and neonatal mortality ratios are 556 per 100,000 live birth and 25 per 1000 live birth respectively [6]. This figure is considerably higher compared with the global rates of 37 per 100,000 for maternal and 17 per 1000 for neonates [7]. The estimated maternal and neonatal mortality ratios are far from Sustainable Development Goals target 3.1 and 3.2 of reaching maternal mortality ratio of less than 70 deaths per 100,000 live births and reduce neonatal mortality to 12 per 1,000 live births and under-5 mortality 25 per 1,000 live births by 2030 [7,8]. The postnatal period is defined as the period from birth to first six weeks post-delivery [9]. It is a critical period for the health and survival of the mother and the newborn. Early postnatal checkup determine early identification and treatment of birth complications [10,11] and give opportunity for early health promotion, early initiation of vaccination, exclusive breastfeeding and other postnatal care services, hence improve the survival of the mother and the newborn [12,13]. The WHO recommends four postnatal visits, the first contact has to happen within the first 24 hours after birth for both home and facility delivery, followed by three additional visits, that is, at 2-3 days, 6-7 days, and at 6 weeks [9].

Similarly, in the efforts to reduce maternal and neonatal death the Tanzania government has adopted the WHO postnatal visit recommendations, that the mother and newborns should receive postnatal contact for at least 24 hours post facility delivery, early as possible within 24 hours for home delivery and continue with three additional postnatal contacts, that is, at 2-3 days, 6-7 days, and at 6 weeks after birth [14]. Other government initiatives to reduce maternal and neonatal death includes ensuring universal health coverage for comprehensive reproductive maternal neonatal and child health (RMNCH), access to and quality of RMNCH, addressing all causes of maternal and neonatal mortality and morbidities, strengthening health systems to collect high quality data, ensure accountability and direct health facility financing [15]. Despite the government efforts, early postnatal care coverage is still low, it is almost 58% of newborns and 66% of mothers did not receive early postnatal care that is within 48 hours after birth [6]. To accelerate the achievement of Sustainable Development Goals target 3.1 and 3.2 [8], and in order to increase early PNC utilization, requires a better understanding of the factors that influence its uptake, both positively and negatively that would be helpful to have targeted interventions. Limited information is available on the factors associated with early postnatal care in Tanzania. Studies using national representative surveys of mothers assessing uptake of early PNC are scarce. Therefore, to fill this gap, this study aims to explore factors associated with early postnatal checkup among women of reproductive age in Tanzania.

## Methods

### Study design

This study was analytical cross-sectional study, conducted using secondary data from the 2015/16 Tanzania Demographic Health Survey (TDHS). The analysis used the women's individual recode data file.

### Study area

The study was carried out in Tanzania. Data were collected across all regions in the country. The country's economy is mostly based on agricultural activities, where women and children are highly involved. About 70% of the population reside in rural areas. Maternal and child health services coverage are improving slowly, antenatal care (ANC), facility delivery and postnatal coverage are 51%, 63% and 37% respectively. However, maternal deaths (556/100,000 live birth) and neonatal mortality (25 per 1000 live birth) in the country are still high compared to global targets [6].

### Study population, sample size and sampling

**Figure 1:**
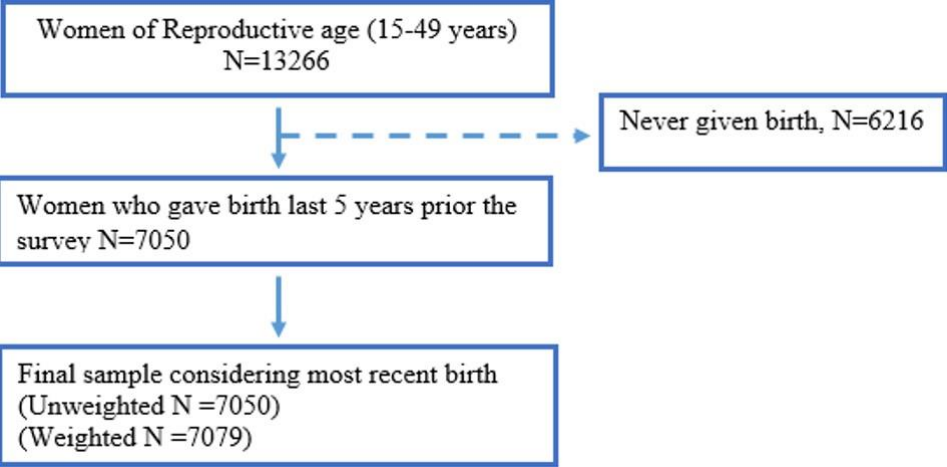
flow chart for selection of study participants

Study population include all women of reproductive age who participated in the 2015/16 TDHS and gave birth 5 years preceding the survey. The analysis was limited to the mother and not the newborn child, where a total of 13266 women of reproductive age were surveyed. After excluding women who did not gave birth five years preceding the survey, a final sample comprised a total of 7050 women were analyzed in this study (Figure 1).

### Study variables

Early postnatal care was the dependent variable in this study, which was defined as having received postnatal checkup by the mother at least once within 48 hours in the health facility. Early postnatal care was coded into a binary variable representing having received early PNC or not. Mothers who had a postnatal checkup within 48 hours of delivery were coded 1. Mothers who did not received postnatal checkup within 48 hours of delivery were coded 0. Therefore ‘0’ group included mothers who received postnatal checkup above 48 hours and mothers who never received a checkup at all. The timing of less than 48 hours was chosen as this is critical time where most of maternal deaths occurs and receiving PNC at this time improve survivals [4]. Independent variables were socio-demographics (place of residence, geographical zones, mother education, age, marital status) and reproductive health characteristics (ANC visits, parity, place of birth, mode of delivery).

### Data management and analysis

STATA software version 15 was used for data analysis, where data was explored for missing values and cleaned. Variables were clearly defined, coded based on the nature of the variable and previous literatures for easier interpretation and comparability between studies. Data quality and privacy was maintained throughout, as the flash and external disc was used for data backup. Data analysis were performed accounting for complex survey nature of the data. Individual women of reproductive age (WRA) were considered a unit of analysis. The analysis used the weighted percentages after accounting for survey weights to ensure national representative estimates for the factors associated with early PNC. Categorical variables were summarized using frequency and proportions while continuous variables were summarized using measures of central tendency and respective measures of dispersion. To determine prevalence of early PNC, descriptive statistics was done, where bar graph showing the prevalence was presented.

To determine the relationship between participants' characteristics and the outcome variable, Pearson's chi square probability test was used. In the crude analysis, the Poisson regression model was modeled to determine the unadjusted prevalence ratios with their corresponding 95% confidence interval. This model was used as an alternative to the classical logistic regression as the proportion of early PNC was above 10% and the log-binomial regression model failed to converge. Statistical significance level was set at p-value of < 0.05. Independent variables with p-value of < 0.05 in crude analysis were entered in the multivariable Poisson regression model to adjust for potential confounding effect. We used stepwise regression for model building whereby covariates were added and removed from the model by backward elimination. The parsimonious model was selected based on the model with the lowest Akaike information criteria (AIC). The crude prevalence ratio (CPR), interquartile ranges (IQR), standard deviation (SD), adjusted prevalence ratio (APR) and 95% confidence interval (CI) were presented. A variable was regarded as a confounder if the changes of the estimate from crude to adjusted analysis > 20%. Both interaction and multicollinearity between exposure variables was assessed. A p-value of less than 5% was considered statistically significant.

### Ethics approval and consent to participate

Ethical approval to conduct this study was obtained from Kilimanjaro Christian Medical University College (KCMUCo) Research and Ethical Committee with clearance number PG.05/2020. Also, the permission to use demographic health survey data was granted by Demographic Health Survey program measure evaluation. Confidentiality in data management, analysis and presentation adhered.

## Results

### Participant’s socio-demographic characteristics

A total weighted sample of 7,079 mothers in the TDHS 2015/16 who reported a birth of a child were included in the analysis, of these 2,548 (36.0%) reported to receive a postnatal checkup within 48 hours post-delivery. Nearly half (42.1%) of participants were aged 25-34 years with the median age of 27 years (IQR 20-36). The majority (70%) of women were from rural areas, where the eastern and lake zones contributed the higher percentages of study participants (16.1% and 28.5% respectively). The majority (64.7%) of participants had attained a primary education. Majority (80.3%) were married, working (78.5%) and reported to have access to media (82.3%). About 39.3% of the mothers reported to be in a higher wealth index versus 41.6% who are poor (Table 1).

**Table 1:** socio-demographic and reproductive characteristics (weighted) of the study participants, Tanzania Demographic Health Survey 2015/16 (N=7,079)

Variables	n	%
**Mother’s age (years)**		
15-24	2314	32.7
25-34	2982	42.1
35-49	1782	25.2
Median (IQR)	27 (20,36)	
**Mother’s education level**		
No education	1350	19.1
Primary education	4580	64.7
Secondary or higher	1149	16.2
**Marital status**		
Never married	515	7.3
Married or partnered	5687	80.3
Divorced/separated	738	10.4
Widowed	137	1.9
**Wealth Index**		
Poor	2947	41.6
Middle	1349	19.1
Richer	2783	39.3
**Place of residence**		
Urban	2123	29.9
Rural	4955	70.0
**Geographical zones**		
Northern	699	9.9
Western	778	11.0
Central	794	11.2
Southern Highlands	426	6.0
Southern	340	4.8
Southwest Highlands	715	10.1
Lake	2015	28.5
Eastern	1137	16.1
Zanzibar	171	2.4
Access to media*		
Yes	5825	82.3
**Occupation status**		
Working	5557	78.5
**Distance to health facility**		
Not a problem	3852	54.4
Age at first marriage (years)*		
15-24	5637	92.7%
25-34	426	7.0%
35-49	17	0.3%
Mean ± SD	18±3.9	
**Place of Delivery**		
Home	2391	33.8
Facility	4688	66.2
Antenatal care attended*		
**Yes**	6902	97
**Parity**		
1	1730	24.4
2-3	2439	34.4
4+	2910	41.1
Median (IQR)	2 (0,4)	
**Mode of delivery**		
Vaginal delivery	6585	93.0
Caesarian section	493	6.9

* Frequencies (n) do not tally to the total due to missing values in these variables

### Reproductive characteristics of the mothers who receive PNC, TDHS 2015/16

Most (92.7%) of the respondents reported to have been married at 15-24 years of age. The mean age (± SD) of first marriage was 18 (± 3.9) years. About a quarter (24.4%) had one child and 41.1% had more than four children. The majority (97%) of women reported to have attended antenatal care, with 50.9% reported to have attended 4 or more antenatal visits. The majority (66.2%) of women reported to have delivered in a facility. Most (93%) had vaginal delivery, 93.7% of the newborn´s weighted 2.5kg and above (Table 1).

### Prevalence of early PNC, TDHS 2015/16

**Figure 2:**
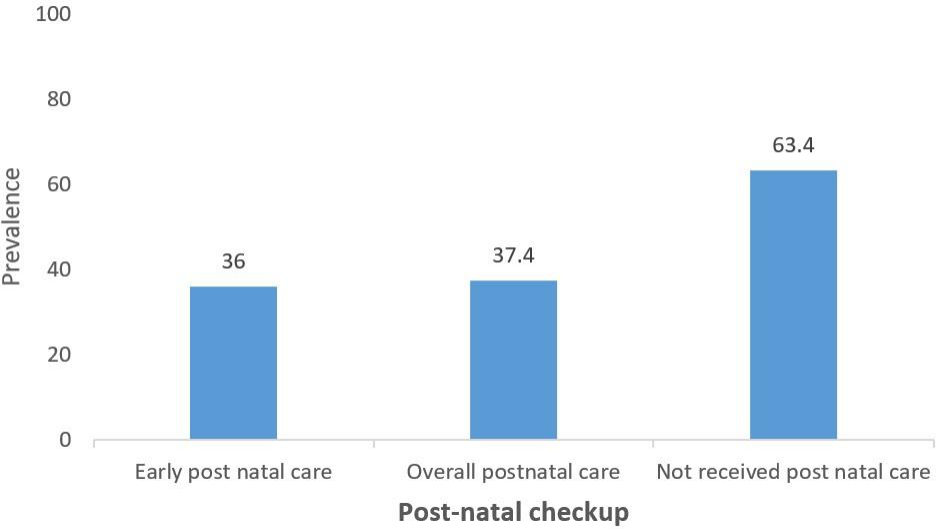
prevalence of early postnatal care (Demographic Health Survey 2015/16, (N=7,079))

The overall prevalence of postnatal care was 37.4%. The prevalence of early PNC was 36.0%. Majority (4445 (63.4%)) received no postnatal care (Figure 2).

### Uptake of early PNC by participant characteristics, TDHS 2015/16

**Figure 3:**
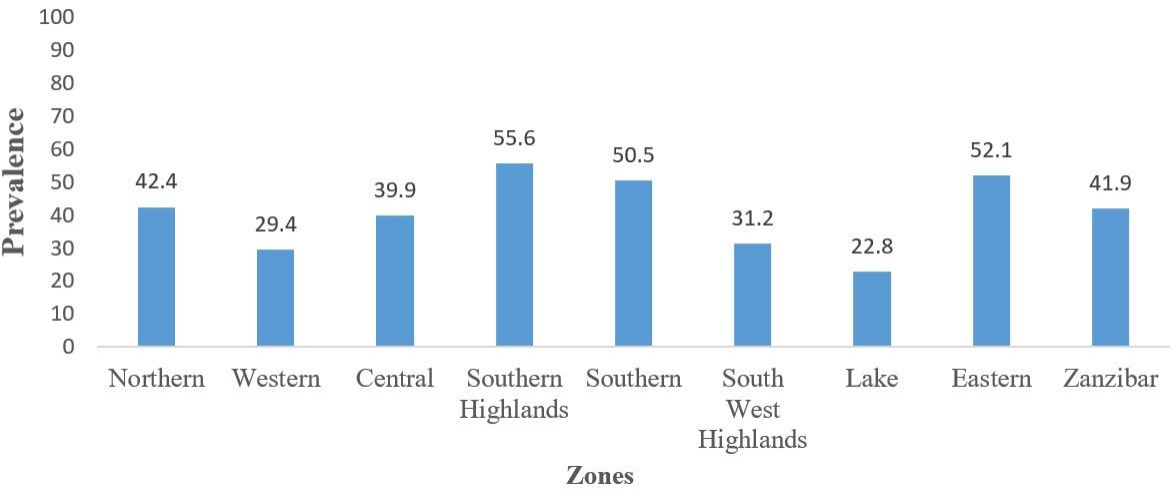
prevalence of early postnatal care by zones (Demographic Health Survey 2015/16, (N=7,079))

The proportional of women who received early PNC according to socio-demographic and reproductive characteristics are shown in Table 2. About half (50.7%) of the respondents who used early PNC were from urban while (30.8%) were from rural areas. The Southern highlands and Eastern zones had a higher prevalence than other zones (55.6% vs 52.1%, respectively), while the Lake zone had the least prevalence (22.8%) of early PNC in Tanzania (Figure 3). The prevalence of early PNC use was significantly higher among women aged 25-34 years (38.9%), who were richer (48.9%), had access to media (39.7%) and to those with secondary education or higher (52.6%) compared to their counterparts. On the other hand, the prevalence of early PNC was low among women who had no ANC visits (15.4%), with home delivery (10.1%), who are working (35.9%), and those whose distance to healthy facility was a problem (32.6%) (Table 2).

**Table 2:** proportion of women (weighted) who achieved an early postnatal care visit, according to socio-demographic and reproductive characteristics, Tanzania Demographic Health Survey 2015/16 (N=7,079)

Variable	Total	Early Post-natal care (n=2586)	p-value
**Place of residence**			<0.001
Urban	2099	1063 (50.7)	
Rural	4944	1522 (30.8)	
**Mother’s age**			0.002
15-24	2308	850 (36.8)	
25-34	2971	1156 (38.9)	
35-49	1767	578 (32.7)	
**Marital status**			0.081
Never married	509	216 (42.6)	
Married or partnered	5667	2070 (36.5)	
Divorced/separated	732	255 (34.8)	
Widowed	137	43 (31.4)	
**Geographical zones**			<0.001
Northern	696	295 (42.4)	
Western	774	227 (29.4)	
Central	794	317 (39.9)	
Southern Highlands	424	235 (55.6)	
Southern	336	170 (50.5)	
Southwest Highlands	713	222 (31.2)	
Lake	2012	460 (22.8)	
Eastern	1123	584 (52.1)	
Zanzibar	171	71 (41.9)	
**Wealth Index**			<0.001
Poor	2943	800 (27.2)	
Middle	1346	435 (32.4)	
Richer	2758	1348 (48.9)	
**Access to media**			<0.001
No	1250	282 (22.6)	
Yes	5796	2302 (39.7)	
**Mother’s education level**			<0.001
No education	1345	338 (25.1)	
Primary education	4560	1647 (36.1)	
Secondary or higher	1142	600 (52.6)	
**Occupation status**			0.056
Not working	1512	598 (39.6)	
Working	5535	1987 (35.9)	
**Place of delivery**			<0.001
Home	2390	240 (10.1)	
Health facility	4657	2308 (49.6)	
**Distance to health facility**			<0.001
Problem	3210	1046 (32.6)	
Not a problem	3837	1540 (40.1)	
**Antenatal care attended**			<0.001
No	139	21 (15.4)	
Yes	6874	2554 (37.2)	
**Parity**			<0.001
1	1722	772 (44.8)	
2-3	2424	975 (40.2)	
4+	2901	838 (28.9)	
**Mode of delivery**			<0.001
Vaginal delivery	6567	2192 (33.4)	
Caesarian section	480	394 (82.1)	

### Factors associated with early PNC, TDHS 2015/2016

In the crude analysis using Poisson regression model, all variables were significantly associated with early PNC use except for occupation status of the participants. The prevalence of early PNC use was 31% (CPR: 0.69, 95%CI: 0.53-0.89) and 47% (CPR: 0.53, 95%CI: 0.44-0.64) lower among mothers in Lake and Southwest zones respectively compared to Northern zone. Women from rural residence had 39% (CPR: 0.61, 95%CI: 0.55-0.67) lower prevalence of early PNC use compared to urban residence. Women with richer wealth index (CPR: 1.79, 95%CI: 1.64-1.97), and those with middle wealth index (CPR: 1.19, 95%CI: 1.05-1.34) had higher prevalence of use PNC within 48 hours compared to women in the poor wealth quintile. Women who had access to media had 76% (CPR: 1.76, 95%CI 1.52-2.03) higher prevalence of PNC use within 48 hours compared to those who did not have access to media. Furthermore, women who had facility delivery (CPR: 4.94, 95%CI: 4.24-5.75) and those with caesarian delivery (CPR: 2.46, 95%CI: 2.29-2.63) had higher prevalence of PNC use compared to their counterparts (Table 3).

**Table 3:** factors associated with early postnatal care in Tanzania (Demographic Health Survey 2015/16)

Variable	CPR (95% CI)	p-value	APR (95% CI)	p-value
**Mother’s age**				
15-24	0.94(0.86- 1.03)	0.162	0.97(0.89-1.07)	0.580
25-34	1		1	
35-49	0.85(0.77- 0.93)	<0.001	0.96(0.88-1.05)	0.391
**Mother’s education level**				
No education	1		1	
Primary education	1.44(1.27-1.62)	<0.001	1.04(0.93-1.18)	0.479
Secondary or higher	2.09(1.81-2.41)	<0.001	1.08(0.93-1.25)	0.316
**Marital status**				
Never married	1		1	
Married or partnered	0.86(0.75- 0.98)	0.023	1.06(0.93-1.20)	0.375
Divorced/separated	0.82(0.69- 0.98)	0.025	0.98(0.83-1.15)	0.790
Widowed	0.75(0.53- 1.06)	0.079	0.98(0.71-1.35)	0.898
**Wealth Index**				
Poor	1		1	
Middle	1.19(1.05-1.34)	0.006	0.93(0.84-1.05)	0.256
Richer	1.79(1.64-1.97)	<0.001	0.94(0.85-1.04)	0.226
**Place of residence**				
Urban	1		1	
Rural	0.61(0.55-0.67)	<0.001	0.91(0.82-0.99)	0.038
**Geographical zones**				
Northern	1		1	
Western	0.69(0.53-0.89)	0.003	0.89(0.75-1.06)	0.203
Central	0.94(0.78-1.13)	0.529	1.09(0.95-1.26)	0.198
Southern Highlands	1.31(1.10-1.55)	0.002	1.14(1.00-1.30)	0.050
Southern	1.19(0.97-1.45)	0.106	1.11(0.93-1.32)	0.256
South West Highlands	0.71(0.56-0.89)	0.003	0.78(0.65-0.93)	0.006
Lake	0.53(0.44-0.64)	<0.001	0.69(0.59-0.79)	<0.001
Eastern	1.21(1.05-1.44)	0.018	0.97(0.85-1.11)	0.656
Zanzibar	0.98(0.83-1.17)	0.812	1.01(0.87-1.16)	0.902
**Access to media**				
No	1		1	
Yes	1.76(1.52-2.03)	<0.001	1.19(1.06-1.34)	0.004
**Occupation status**				
Not working	1			
Working	0.91(0.82-1.00)	0.053	-	
**Distance to health facility**				
Problem	1		1	
Not a problem	1.23(1.13-1.35)	<0.001	1.00(0.93-1.08)	0.948
**Number of antenatal care visits**				
None	1		1	
1-3	1.94(1.18-3.19)	0.009	1.33(0.86-2.07)	0.203
4+	2.78(1.69-4.57)	<0.001	1.50(0.97-2.33)	0.070
**Place of Delivery**				
Home	1		1	
Facility	4.94(4.24-5.75)	<0.001	3.88(3.31-4.54)	<0.001
**Mode of Delivery**				
Vagina	1		1	
Caesarian section	2.46(2.29-2.63)	<0.001	1.67(1.56-1.78)	<0.001
**Parity**				
1	1		1	
2-3	0.90(0.82-0.97)	0.015	0.98(0.89-1.07)	0.646
4+	0.64(0.59-0.71)	<0.001	0.93(0.82-1.04)	0.222

CPR-crude prevalence ratio, APR-adjusted prevalence ratio

In the multivariable analysis, zone, place of residence, access to media, place of delivery and mode of delivery were significantly associated with early PNC after adjusting for other variables. The prevalence of early PNC use was 19% (APR: 1.19, 95%CI: 1.06-1.34) higher among mothers with access to media compared to mothers with no media access. Women who had caesarian delivery had 67% (APR: 1.67, 95%CI: 1.56-1.78) higher prevalence of early PNC use compared to those with vaginal delivery. Furthermore, women who reported having facility delivery had almost 4-fold (APR: 3.88, 95%CI: 3.31-4.54) higher prevalence of utilizing early PNC compared to women with home delivery. Women who reside in rural areas had 9% (APR: 0.91, 95%CI: 0.82-0.99) lower prevalence of utilizing early PNC compared to those who resided in the urban setting. Likewise, the prevalence of early PNC use was lower among mothers in southwest zones and lake zones ((APR: 0.78, 95%CI: 0.65-0.93) versus (APR: 0.69, 95%CI: 0.59-0.79) respectively) compared to those in the Northern zone. There was no significant association between early PNC use with other factors such as; mother´s age, education level, marital status, wealth index, parity, number of antenatal care visits and distance to health facility (Table 3).

## Discussion

The prevalence of early PNC use was 36%. Place of residence, geographical zones, access to media, mode of delivery and place of delivery were significantly associated with early PNC use in our study. The results of early PNC use are higher than the levels reported in other studies in low-income countries [16,17], but lower than the 50% reported in a study in Tanzania, Uganda [18,19] and 48% and 63% reported in Malawi and Zambia respectively [20,21].

The observed findings suggest that a substantial proportion of postpartum mothers do not use early PNC in Tanzania. The low coverage of early PNC in Tanzania can be explained by the lack of awareness of the PNC services [22]. PNC use was regarded as a neglected, poorly used and a weak reproductive, maternal and child health intervention. As a study in South Sudan reported, provision of PNC health education post-delivery were associated with early PNC use [17]. The study shows that mothers with facility delivery were more likely to use early PNC compared to home delivery. Similar findings were reported in recent studies in Tanzania and Uganda [18,23]. The association between facility delivery and early PNC use could be explained by the clinical counselling provided during delivery by the skilled birth attendants. Most of home deliveries are conducted by unskilled personnel, who are unaware of the importance of early checkup and may not educate and encourage the mothers to attend for early PNC at the facility. Contradicting results in other studies showed women who deliver at a health facility did not attend PNC within 48 hours of delivery [19,21]. This finding is counterintuitive because we expected all health facility deliveries would receive early PNC. A plausible explanation for this finding could be, firstly early discharge from the facility too soon after delivery, secondly limited staffing at health facilities and inadequate training of health staff, which constrain provision of early PNC services, and thirdly disrespect and abuse during birth may negatively influencing early PNC use, as has been reported in a study [19].

Among the enabling factors, place of residence was found significantly associated with early PNC. Mothers who lived in rural areas were less likely to receive early PNC compared to urban residents (9% vs 91%). Our finding is supported by a previous study in Ethiopia where urban dwellers were found to utilize more earlier PNC than rural residence [16]. This could be explained by the fact that majority (70%) of our study participants were from rural areas, where there are poor health system. Physical accessibility of public services, such as roads, transport and health services has been previously found to increase maternal health service utilization [24]. Again, cultural malpractice and misconceptions are more prevalent in rural areas these may prevent women from accessing early postnatal care [25]. In the present study, mothers with access to media had higher prevalence of early PNC than those without access to media. This is consistent with previous studies in Zambia and Ethiopia [16,21]. Health promotion through mass media informs and educates the public, hence increases the knowledge and improves individual ability to seek for health care. Women who have access to media are more likely to know the benefits of having PNC after birth, as they are aware of the problems encountered if does not receive PNC [26]. Moreover, this finding may be attributed to the fact that women who have access to media may be more educated and come from wealthier families. As such they are well informed and are able to make decisions on the use of early PNC and other reproductive services. In Tanzania, a number of health adverts from the Ministry of Health are easily and widely accessible through public media with little or no cost. This confirms the importance of health education in enhancing and sustaining use of available health services.

With regard to need factors, our study demonstrated that, the likelihood of early PNC use was higher among women who had caesarian delivery than those who had vaginal delivery. This finding concurs with studies done in Tanzania [19,27] and in other countries [22,28]. It is possible that women who experienced caesarian delivery were provided with closer care and follow-up to ensure a positive outcome. Other factors such as mother´s age, education level, marital status, wealth index, parity, antenatal care visits, and distance to health facility were not statistically significantly associated with early PNC use in the multivariable model. However, these factors are important predictors for early PNC elsewhere [18,20]. Although most of them (education level, marital status, wealth Index, parity, antenatal care visits and distance to health facility) were observed to be significantly associated with early PNC in the bivariate analysis, they failed to achieve significance in the final model, possibly, due to their low prevalence in the sample.

### Strengths and limitations

The strengths of this study are as follows; it has utilized the national representative data, a large sample size, and a household-based survey where it uses a stratified cluster sampling technique which makes our study to have enough power to conclude on the factors associated with early PNC. These also makes the findings generalizable to the whole country, where to the best of our knowledge this is the first study to assess factors associated with early PNC by using country-wide data. Our study makes a great contribution in understanding factors associated with early postnatal checkups for mothers in Tanzania. Also, in the analysis we modeled the multivariable Poisson regression model that help overcome the downfalls of the classical logistic regression when estimating common outcomes which have been used by the previous authors. Despite the strength of our study, there are number of limitations which need to be taken into account while interpreting our results as they might affect our conclusions. The study was limited by the accuracy of the respondent's recall of the timing and receipt of postnatal checks for births up to 5 years prior to interview, however, we only included the respondent´s most recent birth to reduce the extent of the recall error.

Further, as we have used secondary data, we were limited to only variables collected in the TDHS. Factors that may affect early PNC but are not available in TDHS were not examined. For example, the information on the mother's health status, complications surrounding the delivery, and mother's perception of whether there are benefits of postnatal care were not collected. Also, the information on the quality of care provided to the mother during ANC visits, delivery and the content of PNC were not collected. All these factors have been reported to influence early PNC attendance [19,22]. But we were unable to adjust for these factors in the final model therefore, its effect on our results and conclusion remains unquantified. Moreover, being a cross-sectional study, the findings demonstrate only associations with no causal inferences.

## Conclusion

Although early PNC being very important in preventing maternal and neonatal deaths, majority of the mothers in Tanzania did not receive the recommended early postnatal care. Our study demonstrated that, only 36% of the mothers reported to have received postnatal checkup within 48 hours post-delivery. This calls attention to the Ministry of Health leaders and policy makers to scale up early PNC utilization. The results further showed, zone, place of residence, access to media, place of delivery and mode of delivery were important factors associated with early PNC. Therefore, designing interventions that target these factors may increase uptake of early PNC in Tanzania which in turn reduce maternal and neonatal deaths, adding efforts in achieving Sustainable Development Goals target 3.1 and 3.2 to end preventable deaths among mothers and children by 2030.

### What is known about this topic

- *Early PNC is critical for the health and survival of the mother and her child;*
- *Previous study reveals lack of early PNC may lead to death as well as missed opportunities for timely health promotion for the mother and the newborn*.

### What this study adds

- *The study identifies factors associated with early PNC for the mothers in Tanzania who had facility delivery;*
- *The study points out the proportion of mothers who reported to have received postnatal checkup within 48 hours post-delivery*.
